# Colon Stem Cell and Crypt Dynamics Exposed by Cell Lineage Reconstruction

**DOI:** 10.1371/journal.pgen.1002192

**Published:** 2011-07-28

**Authors:** Yitzhak Reizel, Noa Chapal-Ilani, Rivka Adar, Shalev Itzkovitz, Judith Elbaz, Yosef E. Maruvka, Elad Segev, Liran I. Shlush, Nava Dekel, Ehud Shapiro

**Affiliations:** 1 Department of Biological Regulation, Weizmann Institute of Science, Rehovot, Israel; 2 Department of Computer Science and Applied Mathematics, Weizmann Institute of Science, Rehovot, Israel; 3 Department of Biological Chemistry, Weizmann Institute of Science, Rehovot, Israel; 4 Rappaport Faculty of Medicine and Research Institute, Technion and Rambam Medical Center, Haifa, Israel; University of Washington, United States of America

## Abstract

Stem cell dynamics *in vivo* are often being studied by lineage tracing methods. Our laboratory has previously developed a retrospective method for reconstructing cell lineage trees from somatic mutations accumulated in microsatellites. This method was applied here to explore different aspects of stem cell dynamics in the mouse colon without the use of stem cell markers. We first demonstrated the reliability of our method for the study of stem cells by confirming previously established facts, and then we addressed open questions. Our findings confirmed that colon crypts are monoclonal and that, throughout adulthood, the process of monoclonal conversion plays a major role in the maintenance of crypts. The absence of immortal strand mechanism in crypts stem cells was validated by the age-dependent accumulation of microsatellite mutations. In addition, we confirmed the positive correlation between physical and lineage proximity of crypts, by showing that the colon is separated into small domains that share a common ancestor. We gained new data demonstrating that colon epithelium is clustered separately from hematopoietic and other cell types, indicating that the colon is constituted of few progenitors and ruling out significant renewal of colonic epithelium from hematopoietic cells during adulthood. Overall, our study demonstrates the reliability of cell lineage reconstruction for the study of stem cell dynamics, and it further addresses open questions in colon stem cells. In addition, this method can be applied to study stem cell dynamics in other systems.

## Author Summary

The study of stem cell and tissue dynamics *in vivo* is often carried out by lineage tracing methods that depend on the presence of specific markers and on the availability of stem cells. In the current study, we applied a novel method for the reconstruction of cell lineage trees from microsatellite mutations accumulated during mouse life. We focused on the intestinal epithelium, since its stem cells were intensively studied by various tracing methods that clarified many aspects of their dynamics. We first showed the reliability of our method by confirming three previously established facts: the existence of “monoclonal conversion,” the absence of an immortal strand mechanism in colon stem cells, and the separation of the colon into small domains each with a common ancestor. We also answered a few open questions, showing that the colon's lineage is separated from other lineages such as the hematopoietic and pancreatic lineages. Overall, our work presents a new approach for the study of stem cell dynamics and can similarly be used for studying stem cell dynamics in other systems.

## Introduction

Mammalian stem cells and tissue dynamics *in vivo* are presently studied by lineage tracing methods [1], [2], which are dependent on the presence of specific stem cell markers [2]. These methods require generation of transgenic animals, development of sophisticated imaging modalities, and are contingent on the availability of stem cells in a specific tissue [2],[3]. Our laboratory developed a method that utilizes somatic microsatellite (MS) mutations for reconstructing cell lineage trees [4]–[7]. This retrospective method, which was also applied by others [8]–[11], is based on the notion that somatic mutations accumulated during normal cell divisions endow each cell of the body with a genomic signature that, with very high probability, is unique [4]. The distances between the genomic signatures of different cells, as measured using various mathematical methods [12], can then be used to reconstruct the organism's cell lineage tree. In this application of our method, the cellular genomic signature is derived from a set of MS loci in mismatch-repair (MMR) deficient mice (Mlh1−/−). The distance measure used is Maximum Likelihood estimator (Materials and Methods). The MS mutation rate of these mice is much higher than that of wild type, thus increasing the precision of the cell lineage analysis. These mice exhibit normal morphology, but are infertile and develop cancer spontaneously [13]. Up to now, our method was validated using *ex-vivo* cell lineage trees [4] and applied to the lineage analysis of cells of a mouse with a tumor [5]. In addition, it was employed to estimate the number of cell divisions since the zygote, defined as cell depth [6].

The first aim of the present study was to validate the suitability of our method for the study of stem cell and tissue dynamics. We focused on the intestinal epithelium, since its stem cells were intensively studied by various tracing methods that clarified many aspects of their dynamics [2], [14]–[21]. One such aspect, termed ‘monoclonal conversion’, is a process by which intestinal crypts that originate at birth from more than one stem cell, drift toward monoclonality two weeks after birth [22]–[25]. Monoclonal conversion was found to be sustained during mouse life, which means that every few weeks a single stem cell becomes the ancestor of all the cells in the crypt [2], [20], [22]–[27]. Another aspect is the fact that intestinal crypt stem cells do not incorporate an immortal strand [2], [20], [21]. According to the immortal strand hypothesis, stem cells retain the older DNA strand during asymmetric cell divisions and relegate the newly synthesized DNA strand to the differentiated cell, thus avoiding inheritance of mutations caused by DNA replication [28]–[36]. This mechanism was shown to be present in neural stem cells [30]. Other studies suggest its presence in the intestine epithelium stem cells [34], [35]. Most recently, it was shown that there is no asymmetric segregation of DNA within intestinal epithelial stem cells [2], [20], [21], thus making the existence of the immortal strand mechanism, in this system unlikely. However, since this evidence is based on a specific stem cell marker, we still found an additional value in addressing this issue in the intestinal epithelium using our method. Another known result confirmed by our method is the correlation between physical location of crypts and their lineage proximity [37].

Our cell lineage analysis method was applied in the current study not only to validate known results but also to address open questions. Up to date, it was unclear whether during embryogenesis the colon is formed by its own specific progenitors or by cells that are also progenitors of other lineages. In addition, it is well established that during embryogenesis, intestine epithelium cells originate from a lineage different from that of bone marrow cells. However, during adulthood, bone marrow cells were shown to have the capacity to repopulate the gastrointestinal epithelium [38], [39], suggesting that both lineages may interact. Since some doubts were raised regarding the robustness of this process and its relevance to normal physiology [40], we employed cell lineage analysis in the adult mouse to explore clonal relationships between the intestinal epithelium and other lineages, such as the hematopoietic lineage. In order to examine these issues, we applied our method to cells sampled from colonic crypts and other cell types from Mlh1−/− mice at different ages. Our results confirm that ‘monoclonal conversion’ takes place and that intestinal epithelium stem cells do not incorporate an immortal strand. We also confirmed the positive correlation between physical proximity and lineage in colon crypts, and revealed that colon crypts are clustered separately from B-lymphocytes, pancreatic cells (beta and duct cells) and hematopoietic stem cells from the bone marrow. Our findings indicate that the colon is constituted by a few distinct progenitors and that there is no evidence for hematopoietic renewal of the intestinal epithelium during adulthood.

## Results

### Stem cells of the colon epithelium do not retain an immortal strand and undergo constant monoclonal conversion

Our method was first used to confirm that monoclonal conversion occurs in crypt stem cells, and that these cells do not incorporate an immortal strand. Although these facts were already demonstrated by previous studies, deriving this information also from reconstructed cell lineage trees both strengthens these results and establishes the reliability of our method. This validation of our method is followed by the use of the lineage tree reconstruction to generate new information about crypt stem cells.

**Figure 1:**
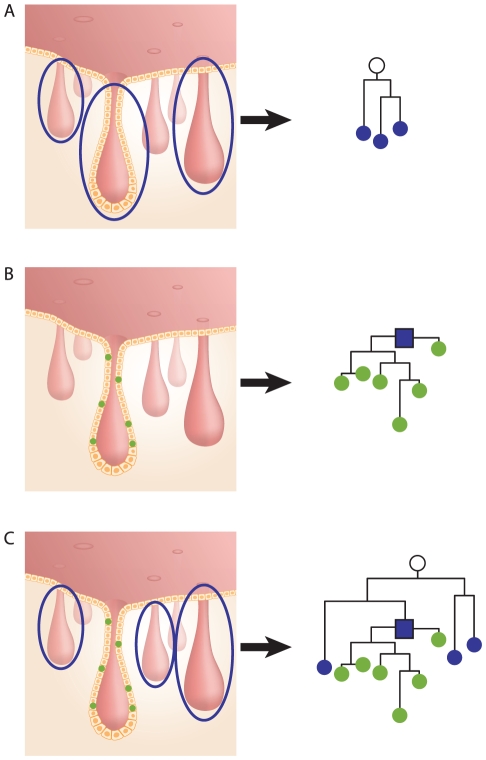
Crypt most recent common stem cell (MRCSC). (A), Left - three whole crypts. DNA extracted from each crypt represents the MCRSC of the crypt. Right - lineage tree of the three crypts' MRCSCs (blue nodes). The root of the tree represents the MRCA (white node) of the three crypts. (B), Left – six individual cells sampled from a single crypt. DNA was extracted from each cell separately. Right - cell lineage tree of the six sampled cells (green nodes). The root of the tree (blue square) represents the crypt's computed MRCSC. (C), Left - DNA was extracted from three whole crypts and from six individually sampled cells of a fourth crypt. Right - the resulting lineage tree shows three crypt MRCSCs (blue nodes), six crypt cells (green nodes), and the computed MRCSC of these cells (blue square). The root of the tree represents the MRCA of the four crypts (white node).

In population genetics, the most recent common ancestor (MRCA) of any set of organisms is the most recent individual from which all organisms in the group are direct descendants. Similarly, we refer to the cell that is the most recent ancestor of all cells in a crypt (stem cells and others) as the crypt's most recent common stem cell (MRCSC). We employ two methods to estimate crypt's MRCSC. One is genotyping the DNA extracted from the entire crypt (all cells in the crypt), considering that the average DNA of all crypt cells is a good approximation of the DNA of the crypt's MRCSC [4] (Figure 1A and Figure S1). Another method utilizes the cell lineage tree reconstructed from DNA extracted from individual cells isolated from a single crypt and refers to the most recent common ancestor node on the tree as the computational MRCSC of the crypt (Figure 1B, blue square).

**Figure 2:**
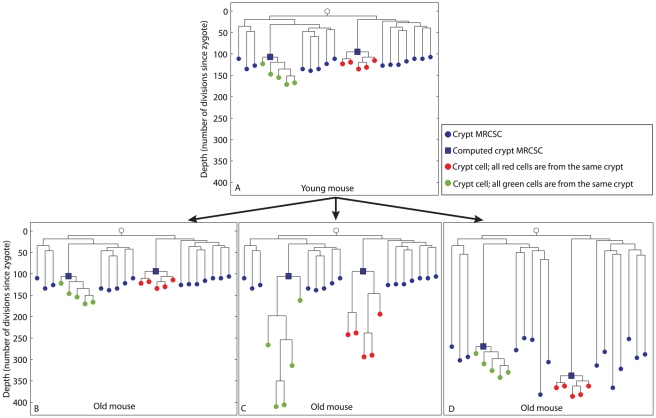
Hypothetical scenarios for intestinal stem cell dynamics. Schematic representation of different possible scenarios of colon crypt dynamics. The Y axis represents cell depth. (A), Hypothetical cell lineage tree of a young mouse. In this tree, crypt MRCSCs (blue nodes) are shallow, individual cells from two crypts (red and green nodes) are separately clustered on the linage tree, and the depth of the computed MRCSC of each crypt (blue squares) is in the same range as the depth of crypt MRCSCs (blue nodes). (B, C, D), In the old mouse we depict three different hypothetical scenarios for stem cell dynamics. (B), Crypt stem cells maintain an immortal strand. As a result, the depth of crypt MRCSCs (blue nodes), crypt cells (red and green nodes) and computed crypt MRCSCs (blue squares) does not change with mouse age. (C), Crypt stem cells do not maintain an immortal strand and there is no monoclonal conversion. Hence all cells of each crypt are the progeny of the stem cell that originated the crypt two weeks after birth. As a result, crypt MRCSCs (blue nodes) and computed MRCSCs (blue squares) do not get deeper with mouse age. However, crypt cells (red and greed nodes) do get deeper when the mouse gets older. (D), Crypt stem cells do not maintain an immortal strand and there is ongoing monoclonal conversion. Hence all cells of each crypt are progeny of a fairly recent single stem cell ancestor regardless of mouse age. As a result the depth of crypt MRCSCs (blue nodes) and of crypt cells (red and green nodes) and their computed MRCSCs (blue squares) increase dramatically with mouse age.

Knowing the topology of the reconstructed cell lineage trees can help understand the history and dynamics of crypt stem cells, as illustrated by Figure 2. The top of the figure presents the hypothetical reconstructed lineage tree of crypt cells and whole crypts sampled from a young mouse. It is well established that young mouse crypts are monoclonal [2], [20], [23]–[25], therefore, in this tree individual cells randomly isolated from two crypts are separately clustered on the lineage tree (red and green nodes, Figure 2A). In this young mouse the latest monoclonal conversion event in a crypt must have occurred only a few cell divisions earlier [2], [20], [24], therefore individual crypt cells are only slightly deeper than their computed MRCSCs.

The trees at the bottom panel of Figure 2 that were qualitatively drawn by us, represent different hypothetical scenarios as the mouse gets older; to illustrate how different biological scenarios may give rise to different cell lineage trees and, conversely, that a biological scenario can be inferred from the structure of the cell lineage tree. According to the first scenario (Figure 2B), crypt stem cells retain an immortal strand [30], [32]–[36]. In that case, they do not accumulate mutations during cell divisions and therefore the depth (number of cell divisions since the zygote) of crypt MRCSCs, depicted as blue nodes, and the depth of computed MRCSCs (blue squares) do not increase with mouse age. Due to the constant depth of crypt MRCSCs, the depth of crypt cells (red and green nodes) does not change with mouse age. According to the second hypothetical scenario (Figure 2C), crypt stem cells do not retain an immortal strand. Thus, these stem cells accumulate MS mutations with mouse age. In addition, crypt stem cells undergo monoclonal conversions only once during their lifetime (2 weeks after birth), and maintain the crypt during growth and adulthood solely due to asymmetric divisions. Therefore, although crypt stem cells underwent many cell divisions and accumulated numerous MS mutations, they are all descendants of the original ancestor (crypt MRCSC) that constituted the crypt when it was young. In the resulting cell lineage tree, crypt MRCSCs (blue nodes) and computed MRCSCs (blue squares) do not get deeper with mouse age. However, individual crypt cells (red and green nodes) do get deeper with mouse age since the stem cells that maintain the crypt accumulate somatic mutations during asymmetric cell division. According to the third scenario (Figure 2D), crypt stem cells do not retain an immortal strand either, but they undergo symmetric cell divisions that lead to constant monoclonal conversions throughout adulthood [2], [20], [23]–[25]. Thus in the cell lineage tree, similarly to the second scenario (Figure 2C), crypt cells (red and green nodes), do get deeper with mouse age due to the accumulation of MS mutations in crypt stem cells. However, unlike the second scenario, crypt MRCSCs (blue nodes) and computed crypt MRCSCs (blue squares) increase dramatically with mouse age, since, over time, crypt monoclonal conversion causes each crypt to become the progeny of a single, fairly recent, stem cell. Recent data support this third scenario [2], [20], [21].

Our method was applied to decipher which of the above hypothetical scenarios holds. For this purpose, whole colon crypts as well as individual crypt cells were isolated by tissue digestion from young and old mice (52 and 340 day-old).

**Figure 3:**
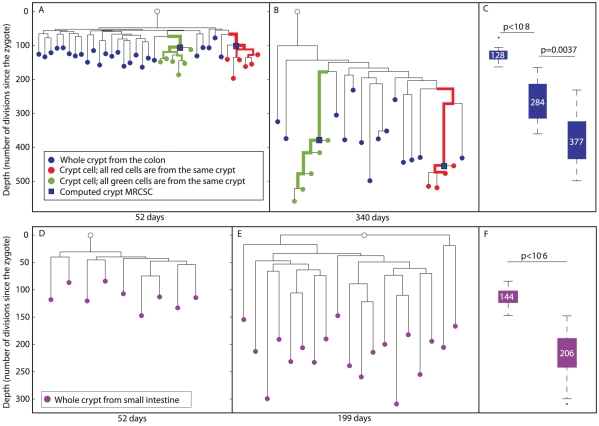
Reconstructed cell lineage trees show stem cell dynamics in colon and small intestine. (A and B), Two reconstructed cell lineage trees of colon cells of 52 and 340 day-old mice, respectively. In the tree, DNA extracted from whole crypts is represented by blue nodes, single cells isolated randomly from an individual crypt by red and green nodes, and the computed crypt MRCSCs are represented by blue squares. Bold branches represent statistically enriched branches for cells from the same crypt. The width of the branches represents clustering significance (wider = lower p value). The Y axis measures cell depth. (C), Box plots of the depths of whole crypts in 52, 199 and 340 day-old mice. Crypt depth increases significantly between 52 and 199 day-old mice (p<10^−8^) and between 199 and 340 day-old mice (p<0.002). Y axis is the same as in A and B. The median depth is shown in each box plot. (D and E), Two reconstructed lineage trees of small intestine cells of 52 and 199 day-old mice, respectively. In the tree, DNA extracted from whole crypts is represented by purple nodes. The Y axis measures cell depth. (F), Box plots of the depths of whole crypts of 52 and 199 day-old mice. Crypt depth increases significantly between 52 and 199 day-old mice (p<10^−6^). Y axis is the same as in D. The median depth is shown in each box plot.

On the reconstructed cell lineage tree, single cells are represented as red and green nodes, whole crypts as blue nodes and computed crypt MRCSCs as blue squares (Figure 1 and Figure 3). We noted that the lineage tree reconstructed from an old mouse agreed with the third hypothetical scenario (Figure 2D), indicating that crypt stem cells do not retain an immortal strand but do undergo constant symmetric cell divisions throughout adulthood leading to monoclonal conversions.

For a deeper analysis of the resulting cell lineage trees, several parameters were examined quantitatively. This analysis revealed that in both young and old mice, individual cells randomly isolated from the same crypt were significantly clustered separately from all other cells (p<10^−7^ for both red and green crypts of the young mouse, p<10^−5^ for the green crypt of the old mouse, and p = 0.0006 for the red crypt of the old mouse, Figure 3A and 3B). In addition, since MS mutations occur randomly and independently during cell division, and since there is great similarity between the genomic signatures of individual cells isolated from the same crypt, we conclude that each analyzed crypt is monoclonal with very high probability. We further examined the depth of whole crypts and single cells isolated from different crypts, assuming that the average mutation rate of MS in our system is about 1 to 100 cell divisions. This estimation is based on the already known division rate of crypt stem cells (Materials and Methods). Our analysis revealed that the depth of colon whole crypts increases significantly and linearly (r^2^ = 0.99) with mouse age (Figure 3C). The median depth of whole crypts (blue nodes) at 52, 199, and 340 days is 128±4, 284±18, and 377±20 cell divisions, respectively. This total increase of about 250 cell divisions during 288 days is very close to the estimation in the literature [2]. In addition, we found that individual crypt cells become significantly deeper with mouse age (Kolmogorov-Smirnov, p<10^−5^), from 148±7 cell divisions in 52 day-old mouse to 478±19 cell divisions in 340 day-old mouse (Figure S2). Since the increase in depth with mouse age demonstrates that crypt stem cells accumulate MS mutations, these findings rule out the presence of an immortal strand.

We define the depth of a crypt cell relative to the crypt's MRCSC to be the number of cell divisions that separate the cell from the crypt's MRCSC. We measured the depth of randomly sampled individual crypt cells (Figure 3A, red and green nodes) relative to their computed MRCSCs (blue squares) and found no statistically significant difference in this relative depth between young and old mice (Figure 3 and Figure S3). The fact that the number of cell divisions that occur in crypt cells since their MRCSCs is independent of mouse age confirms that monoclonal conversion occurs at the same rate independently of mouse age. Moreover, most crypt cells have a depth of about 45 cell divisions relative to their computed MRCSCs (Figure S3), which is very close to the published data [2].

Lastly, the average branch length between whole crypts (blue nodes) to the MRCA of whole crypts was examined. We found that this length significantly increases with mouse age, from an average length of 56±3 to 191±23 cell divisions in 52 and 340 day-old mice, respectively (Figure 3A and 3B, Kolmogorov-Smirnov p<10^−5^). This finding indicates that each crypt underwent an independent cell division process in the colon.

In order to confirm that the depth of computed crypt MRCSCs of individual cells from the same crypt (blue squares) is reliable, we examined whether it is similar to the depth of whole crypts (blue nodes). Our data show that these depths do not differ significantly. Specifically, the range of depths of whole crypts in the 52 day-old mouse was from 78 to 164 cell divisions, and that of the computed crypt MRCSCs of cells from the same crypt depths were 99 and 106. The range of whole crypt depths of the 340 day-old mouse was from 232 to 499, and that of computed crypt MRCSCs were 380 and 455. This observation shows that computed crypt MRCSCs depth is similar to that of whole crypts, thus validating the reliability of depth estimation of internal branches.

We checked for PCR noise by repeating the biochemical analysis of the same biological sample. This analysis revealed that repeat pairs are very close to each other in the lineage tree (Figure S4). This was true for both, DNA extracted from whole crypts and for DNA extracted from single cells, eliminating the possibility that the topology of the tree as well as depth estimation is influenced significantly by PCR noise.

### Stem cells of the small intestine epithelium do not retain an immortal strand and undergo constant monoclonal conversion

According to the literature, stem cells of the small intestine (SI) epithelium are similar to those of the colon in the sense that they do not retain an immortal strand and undergo constant monoclonal conversion [2], [20], [21]. We examined whether the reconstructed trees of the SI are similar to those of the colon. For this purpose, whole SI crypts were isolated by tissue digestion from 52 and 199 day-old mice. The median depth of whole SI crypts (purple nodes, Figure 3) was 114±7 cell divisions at 52 days and 206±10 at 199 days (Figure 3D–3F). This indicates that about 92 cell divisions took place during 147 days (Kolmogorov-Smirnov p<10^−6^). The increase in depth with mouse age demonstrates that crypt stem cells accumulate MS mutations ruling out the presence of an immortal strand in these cells.

In addition, a significant increase with mouse age of the depth of whole crypts relative to the MRCA was observed. This relative depth increased from an average of 65±5 cell divisions in a 52 day-old mouse to an average of 137±9 in 199 day-old mouse (Kolmogorov-Smirnov p<10^−6^). This indicates that each small intestinal crypt underwent an independent cell division process. It is important to note that depth increase does not differ in a statistically significant way between crypts in the colon and the SI.

### Colon phylogeography

**Figure 4:**
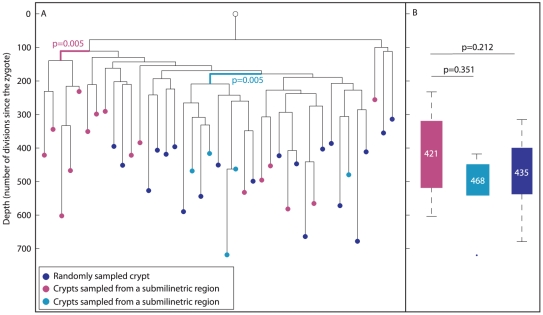
Phylogeography of the colon. (A), In a reconstructed lineage tree, colon crypts isolated by laser capture from submilimetric regions are clustered separately than crypts that were sampled randomly. The tree shows two groups of colon crypts isolated from two different submilimetric regions (cyan and purple) and randomly sampled crypts (blue) isolated from a 278 day-old mouse. Bold branches represent statistically enriched branches for colon crypts sampled from submilmetric regions in the colon. The most significant p value is indicated. The Y axis measures cell depth. (B), Median depths of randomly and submilimetric sampled crypts. Box plots of the depths of submilimetric (cyan and purple) and randomly (blue) sampled crypts. Y axis is the same as in A. The median depth is shown in each box plot.

To study the correlation between the location and lineage proximity of colon crypts, we randomly sampled colon crypts from longitudinal sections by laser capture (Figure 4A, blue nodes). In addition, adjacent crypts were sampled from two small regions (smaller than 1 mm) in the colon (cyan and magenta, Figure 4A). It can be seen that crypts that were sampled from submilimietric regions were significantly clustered on the lineage tree (p = 0.005) in contrast to randomly sampled crypts (Figure 4A), confirming the positive correlation between physical and lineage proximity [37]. Figure 4B indicates that there is no statistically significant difference in depth between crypts isolated from different regions.

Overall, the above findings indicate that conclusions can be drawn reliably from analyzing reconstructed cell lineage trees in the context of colon stem cell dynamics.

### Colon epithelium lineage is different from that of hematopoietic and pancreatic cells

**Figure 5:**
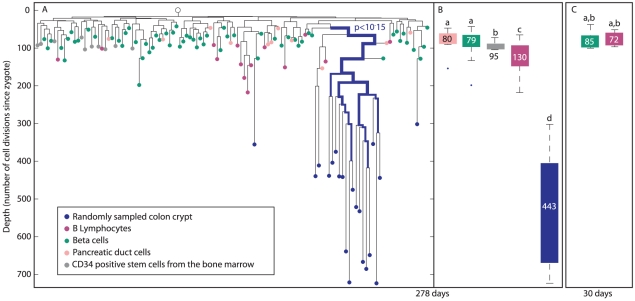
Colon crypts are clustered differently than B-lymphocytes, beta cells, and pancreatic duct cells. (A), A reconstructed lineage tree of colon crypts (blue), B-lymphocytes (purple), beta cells (green), CD34 positive cells from the bone marrow (gray), and pancreatic duct cells (pink) in a 278 day-old mouse. Bold branches represent statistical enrichments on the lineage tree. The most significant p value is indicated. The width of the branches represents clustering significance (wider = lower p value). The Y axis represents depth. (B), Box plots of the depths of crypts (blue), B-lymphocytes (purple), beta cells (green), pancreatic duct cells (pink), and CD34 positive cells from the bone marrow (gray) of a 278 day-old mouse. (C), Box plots of the depths of beta cells (green) and B-lymphocytes (purple) of a 30 day-old mouse. (B and C), Y axis is the same as in A. The median depth of each cell type is shown in the box plots. Columns with different superscripts differ significantly (p<0.05), e.g. pancreatic beta cells depth is statistically significantly shallower than that of B-lymphocytes, and therefore pancreatic beta cells are marked with ‘a’ and B-lymphocytes with ‘c’.

Colon crypts from a 278 day-old mouse were randomly sampled using laser capture microdissection (blue nodes, Figure 5A). In addition, cell types such as pancreatic duct cells (pink), CD34 positive hematopoietic stem cells from the bone marrow (gray), B-lymphocytes extracted from the spleen, thymus and lymph nodes (purple) as well as beta cells extracted from different islets of Langerhans (green) were isolated. The linage tree of these cells was reconstructed using the above mentioned algorithm. We examined whether different colon crypts are clustered separately on the lineage tree, by testing whether crypts are enriched within a given cell population (Materials and Methods). Such clustering of a cell population would suggest a small number of embryonically distinct progenitors. We found that randomly sampled colon crypts are clustered separately on this lineage tree (p<10^−15^, Figure 5A), indicating that only few distinctive progenitors generated this tissue.

### The depth of pancreatic beta cells does not increase with age while that of B-lymphocytes increases less than colon epithelium

Colon crypts isolated from 278 day-old mouse are substantially deeper than all other cell types, including B-lymphocytes that are known to proliferate throughout adulthood (Figure 5B). While the median depth of colon crypts in this mouse is 430±30, the median depth of B-lymphocytes, CD34 positive cells from the bone marrow, beta cells and pancreatic duct cells is 130±11, 95±3, 79±3, 80±9, respectively. Interestingly, each of these cell types has a narrow depths distribution indicating a low standard error. Thus each of these cell types has a characteristic depth range. We noted that CD34 positive cells from the bone marrow, which are the founder population of B-lymphocytes, have much shallower and narrower distribution of depths than B-lymphocytes. In 30 day-old mouse, B-lymphocytes and beta cells depth is 73±3 and 85±6, respectively (Figure 5C). Therefore, we estimate that B-lymphocytes divide every 4.5 days and pancreatic beta cells depth does not increase significantly with mouse age.

## Discussion

### Reliability of the cell lineage reconstruction method

Our study shows that reconstructed cell lineage tree obtained by the analysis of a few dozen MS loci per single cell in Mlh−/− mouse is sufficient to provide reliable information regarding stem cell dynamics. This conclusion is based on the fact that our trees deliver information that is consistent with well-established facts related to colon stem cells.

First, our observation that single cells randomly sampled from the same crypt are always clustered on the trees indicates that our method enables the detection of monoclonality as well as the distinction between separated lineages in nature. Second, the elongation of whole crypt branches with mouse age shows that reconstructed cell lineage trees may demonstrate that in the colon, each crypt develops in an independent manner. Therefore, branch length may serve as a tool to detect stem cell dynamics. Third, the distance between single cells isolated from the same crypt to their computed MRCSCs was about 40 cell divisions, which is in accordance with the literature. This supports the accuracy of depth estimation of internal branches. Fourth, in accordance with the literature we found that adjacent crypts were clustered on the lineage tree, which shows that the colon is separated into small domains that share a common ancestor [37]. Phylogeography analysis could be applicable to many other tissues.

Finally, the mutation rate used in our system was calibrated according to the division rate established in the colon which is one cell division per day [2] (Materials and Methods). The mutation rate obtained from this calibration was applied to the SI and B-lymphocytes, resulting in depths estimation that agrees with that described in the literature [2], [20], [42].

### Colon crypts are constituted by a few progenitors

The observation, that colon cells were enriched separately from B-lymphocytes and CD34 positive cells from the bone marrow as well as from other cell types, shows that this lineage is constituted by few progenitors. In addition, it indicates that unlike pathological conditions which allow the penetration of hematopoietic cells in order to reconstitute the intestine epithelium [38], during normal physiology bone marrow cells do not significantly renew the intestine.

### Applicability of the cell lineage reconstruction method

In this study, we validated many aspects of colonic stem cell dynamics which are already known. Once established, our method may give new insights about healthy and pathologic tissues, in which many aspects of stem cell dynamics are still debated or unknown. The lack of information in these tissues could result from the absence of specific stem cell markers or from the low availability of these stem cells. The topology of reconstructed cell lineage trees can overcome these limitations and expose many aspects of stem cell dynamics. In the eye epithelium for example, due to the lack of specific stem cell markers, the lineage relationship between the cornea and the conjunctiva is still under debate. Specifically, it is not clear whether during adulthood, conjunctiva and corneal cells originate from the same or different stem cells [43], [44]. The topology of the cell lineage tree may answer this debate. Enrichment of conjunctiva and corneal cells on separate branches would indicate that these cells compose two separated populations, while intermingling of these two populations on the tree would reveal that there is no lineage barrier between them.

Overall our cell lineage reconstruction method shows the power of using somatic mutations to decipher developmental and physiological features in crypts and stem cell dynamics. This could be applicable to a wide range of other tissues and stem cells.

## Materials and Methods

### Animals

C57Bl/6 mice, Mlh1+/− (kind donation of Prof. Michael Liskay) [13] and 129SvEv mice, Mlh1+/− (kindly provided by Prof. Ari Elson from the Weizmann Institute, Israel) were mated to yield Mlh1−/− progeny of the dual backgrounds, enabling us to distinguish, in all our experiments, between two alleles in the same locus. All animal husbandry and euthanasia procedures were performed in accordance with the Institutional Animal Care and Use Committee at the Weizmann Institute of Science.

### Digestion of whole crypts and single cells' isolation

Animals were sacrificed before colon isolation. The colon was then sliced into small pieces and incubated at 37°C in Hanks balanced salt solution (HBSS, Sigma Aldrich) containing 0.5 mM EDTA (Sigma Aldrich). After 30 min, the tissue was removed from the medium into a glass tube containing 5 ml HBSS, and stirred for 15 min followed by 2 min centrifugation at 900 RPM. The supernatant was discarded, and the remaining cells were fixed in 70% ice cold ethanol. Single crypts were isolated under the microscope. To isolate single cells from a crypt, each crypt was incubated separately at 37°C for 5 min in a medium containing 0.025% pepsin at pH 2, followed by tiny needle disassembly into single cells. Aliquots of 0.5 µl were spread on a flat bottom 96 well plate (costar 3596, corning) and observed under the microscope. Drops that contained single cell were collected into 0.2 ml tubes and subjected to whole genome amplification.

### Preparation of tissue sections

Frozen mouse tissues were cut at −20°C into 9 µm sections using a cryostat microtome (CRYOTOME – LEICA CM3050 S) and mounted on membrane-coated slides (PALM MembraneSlides – 1 mm PEN membrane covered, PALM Microlaser Technologies). Tissue sections were stained with Hematoxylin and Eosin solutions (Sigma Aldrich) according to the following protocol: 1 min in 70% ethanol followed by several rinses in double-distilled water (DDW), 30 sec in Hematoxylin, 2 min in tap water pre-filtered with 0.2 µm disposable filter units (Schleicher & Schuell), several brief rinses in Eosin, several rinses in 70% ethanol and several rinses in 100% ethanol. Following staining, tissue sections were dried for 5 min at room temperature prior to laser micro dissection.

### Laser-assisted micro-dissection

As previously demonstrated [45], laser micro dissection was performed using the PALM MicroBeam micro-dissection apparatus (PALM Microlaser Technologies). Parameters for laser energy, focus, and speed were adjusted individually for every tissue section, such that dissection was performed with minimal laser energy. The minimal energy level was determined by performing continuous laser micro dissection with decreasing energy levels on a portion of the section adjacent to the area destined for cell isolation. Single cell samples were catapulted using default catapulting energy and focus parameters into adhesive caps of 0.2 ml micro-tubes (PALM Microlaser Technologies). In order to verify successful catapulting, the single cells were subjected to whole genome amplification followed by a preliminary PCR over a panel of 16 microsatellite loci (out of 120).

### Isolation of CD34 positive cells from bone marrow

Bone marrow cells were harvested by flashing the marrow with PBS. Cells were frozen in 90% fetal calf serum (Beit Haemek, Israel) and 10% DMSO (Sigma Aldrich). Prior to FACS analysis bone marrow cells were thawed and washed twice with PBS. Cells were stained with Anti CD34-pacific blue antibody (eBioscience), and sorted by FACS ARIA. CD34 positive cells were separated to single cells by serial dilutions and microscopic observation as described above.

### Whole-genome amplification (WGA) and PCR of single cells

WGA was performed using the Illustra GenomiPhi V2 DNA Amplification kit (GE Healthcare Life Sciences) according to the manufacturer's optimized instructions [46]. Briefly, single cells were picked up from a 96-well, flat bottom plate using 3 µl sample buffer from the kit and transferred to 0.2 ml PCR tubes. Cell lysis, 10 min at 30°C, was done by adding to each tube 1.5 µl cell lysis solution (600 mM KOH, 10 mM EDTA, 100 mM dithiothreitol (DTT)), followed by the addition of 1.5 µl neutralizing solution (4 vol 1 M Tris-HCl, pH 8.0, added to 1 vol of 3 M HCl). WGA, 4 h at 30°C, was initiated by adding 14 µl mix composed of: 4 µl sample buffer, 9 µl reaction buffer, and 1 µl enzyme mix, all supplied with the kit. The reaction was terminated by heat inactivation at 65°C for 10 min. The resulting product was diluted 1∶20 in DDW and analyzed, without any further purification, by PCR, on a preliminary panel of 16 microsatellite loci. Positive cells were further tested on 120 MS loci panel (Table S1). It is important to note that many of the loci we analyzed are of the X chromosome and since in this work we used only male mice, we were able to receive loci with only one allele, thus avoiding, in these loci, the appearance of two alleles with the same length. PCR repeats and negative controls (DDW) were included in every PCR plate. Loci that exhibit a signal in the negative control were excluded from the analysis of all samples run on the corresponding PCR plate. Signal to noise ratio, introduced by the PCR amplification has been assessed for each tree (Figure S4).

### Tree and depth reconstruction

MS length was analyzed based on the capillary signals received by the 3730xl DNA Analyzer. Capillary signals that displayed more than one allele per locus were removed from the analysis. Only cells in which more than 25 alleles were amplified were included in the analysis. The size of each allele was determined, providing a genomic signature - the deviation of MS repeats at each locus from the putative zygote. The signatures were used to reconstruct lineage trees by Neighbor Joining algorithm [47]. Each entry in the distance matrix was taken as the maximum likelihood of the number of divisions separating the two cells, given the observed mutational distance between them. The mutational model assumed in the maximum likelihood approach is a multistep model including only insertion and deletion of one or two repeats in each mutation event. This model showed the best description of the *ex-vivo* trees (Chapal-Ilani et. al., unpublished results). The step probability function was estimated from *ex vivo* trees to be 7∶1 for the single step mutation. We also assumed that the probability of insertion and deletion is equal.

In order to reconstruct a cell lineage tree with an accurate topology, a reliable estimation of the average mutation rate acquired per cell division is necessary, as it enables the conversion of relatively acquired mutations into an absolute number of cell divisions since the zygote (depth). The estimation was executed by calibrating it to the known division rate of colon stem cells during adulthood, which is about one cell division per day [2]. Therefore, the number of cell divisions of mouse colon stem cells at a certain age is equal to its age plus the number of divisions that occurred during the embryonic period, which is known to be between 1–3 cell divisions per day. Thus, the difference in number of divisions between old and young mouse should be the difference between their ages at sacrifice. The estimation of the average mutation rate (including mutation of one step or two steps together) per cell division according to this calibration is 1/100. This estimate is lower than the estimate we derived for cells dividing *in vitro*, 1/42 [6], probably due to the differences between the *in vivo* and *in vitro* systems.

Depth was calculated from the trees as the branch lengths leading from the root to each terminal leaf. Root signature was taken as the allele size values of tail cells [6]. Since the tail contains cells that originate from ectoderm, endoderm and mesoderm, its genomic signature represents the zygote, or one of its immediate descendants. A full description of the product length of each of the sampled cells is presented in Table S2, and the mutation distribution of each of the cell types in the different animals is shown in Figure S5.

### Statistical analysis

P-values for the difference in distributions of cell depths were calculated using the Kolmogorov-Smirnov 2 parameters test.

Hypergeometric tests were carried out in order to detect a significant clustering of a predefined group of cells on the reconstructed lineage tree. According to the method, given a dichotomous classification of 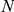 cells in an experiment where 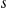 cells belong to group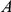 and 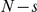 cells belong to the complementary group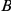, for every branch/internal node in the inferred lineage tree, the null hypotheses of no association between the sub-tree and the classification is tested. This is done by performing a hypergeometric test. Given a subtree of 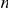 cells in which 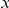 cells are of type 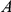 , the branch p-value is the probability to see 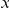 or more cells of type 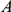 given that the 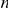 cells are random samples from 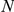:
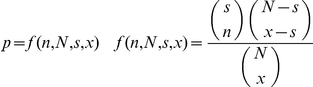
We used a False Discovery Rate correction with an FDR of 20% to determine the p-value threshold for the tree in order to take into account the multi hypotheses, from the fact that there are many sub-trees.

## Supporting Information

Figure S1 Illustration of the median genomic signature of randomly sampled crypt cells as an approximation of the genomic signature of the crypt's MRCSC. (A), Cell lineage tree of six randomly sampled crypt cells (red nodes). The root of the tree (blue square) represents the crypt's computed MRCSC (as shown in Figure 1). (B), Example of few MS loci signatures of the six sampled cells and the computed crypt's MRCSC. The mutation sizes of the different loci in each cell are the deviation of their repeats from the zygote. The signature of the computed MRCSC is the median signature of the six sampled cells.
(TIF)

Figure S2 Depth of individual crypt cells increases with mouse age. Box plots of the depths of single cells isolated from 52 and 340 day-old mice, as depicted in the trees of Figure 3A and 3B. Single cells depth increases significantly between 52 and 340 day-old mice (p = 10^−8^).
(TIF)

Figure S3 No statistically significant difference in relative depth, between young and old mice. Box plots of the relative depths between individual crypt cells to their computed MRCSCs in 52 and 340 day-old mice. There is no statistically significant difference in relative depth between 52 and 340 day-old mice.
(TIF)

Figure S4 PCR repeats are clustered on the reconstructed lineage tree. Reconstructed lineage tree of 52 and 340 day-old mice (Figure 3A and 3B), shows that PCR repeats (red) are strongly clustered and share similar depths. Similar results were received in all trees.
(TIF)

Figure S5 Mutation size frequencies in different cell types from different mice. (A), Mutation size frequencies of whole crypts and single cells isolated from the colon of 52 and 340 day-old mice. (B), Mutation size frequencies of whole crypts isolated from small intestine of 52 and 199 day-old mice. In both A and B, the mutation step size distribution is much wider in the older animal, showing positive correlation between number of cell divisions and mutation lengths. (C), Mutation size frequencies of different cell types from 278 day-old mouse. The distribution of the colon crypts is the widest among all the cell types presented in this panel, since the colon crypts accumulated more MS mutations.
(TIF)

Table S1 Microsatellites panel. Description of the microsatellite loci used in our experiments. The table contains the loci names, their size and forward and reverse sequences. Loci name (first column): [Organism type][Chromosome]_[Basic unit][number of repeats]_[serial number]. Organism type is M for mouse; Chromosome can be a number or the letter X.
(XLS)

Table S2 Fragment sizes. The table contains information of each analyzed cell. This includes the name of the animal it was taken from (Animal ID) the name of the sample (Sample ID), a description of its origin (Tissue), and the isolation method used. In addition, it contains the sizes of the fragments of the different loci as calculated from the 3730xl DNA Analyzer histograms. Each locus size is depicted in different column and the name of the locus is presented in the header line. For X chromosome loci, only the size of one allele is presented (all our samples were taken from male mice), while for all other loci the sizes of both alleles are presented (each one in different column). ‘X’ represents missing data and ‘Null’ indicates that the specific locus was not measured for the sample.
(XLS)
