# Supplementary figures and images for: Colon Stem Cell and Crypt Dynamics Exposed by Cell Lineage Reconstruction
Source: PLoS Genet. 2011 Jul 28;7(7):e1002192. doi: 10.1371/journal.pgen.1002192 (PMC3145618; doi:10.1371/journal.pgen.1002192)

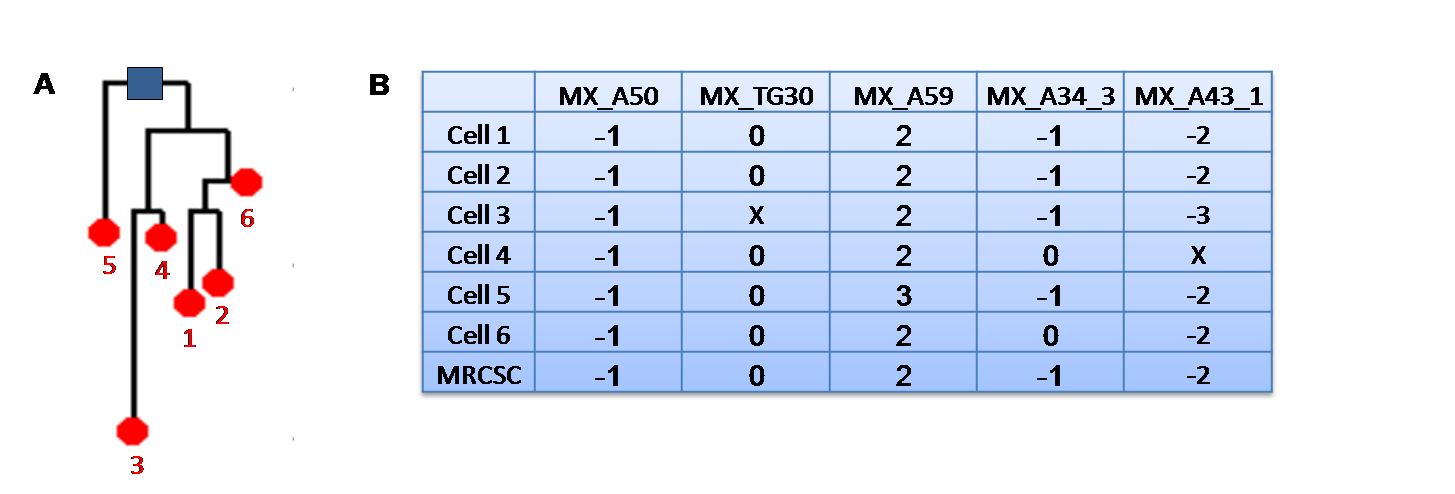

Supplement: Figure S1 — Illustration of the median genomic signature of randomly sampled crypt cells as an approximation of the genomic signature of the crypt's MRCSC. (A), Cell lineage tree of six randomly sampled crypt cells (red nodes). The root of the tree (blue square) represents the crypt's computed MRCSC (as shown in Figure 1). (B), Example of few MS loci signatures of the six sampled cells and the computed crypt's MRCSC. The mutation sizes of the different loci in each cell are the deviation of their repeats from the zygote. The signature of the computed MRCSC is the median signature of the six sampled cells. (TIF) [file pgen.1002192.s001.tif]

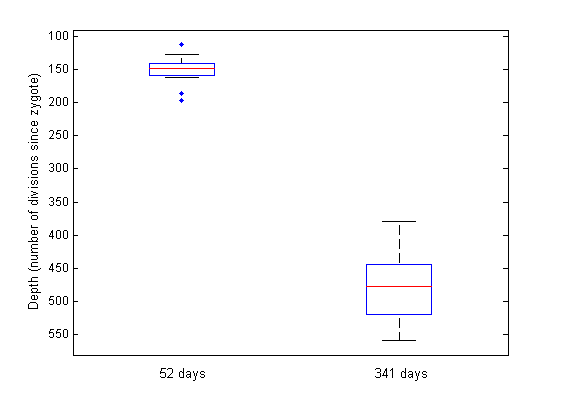

Supplement: Figure S2 — Depth of individual crypt cells increases with mouse age. Box plots of the depths of single cells isolated from 52 and 340 day-old mice, as depicted in the trees of Figure 3A and 3B. Single cells depth increases significantly between 52 and 340 day-old mice (p = 10−8). (TIF) [file pgen.1002192.s002.tif]

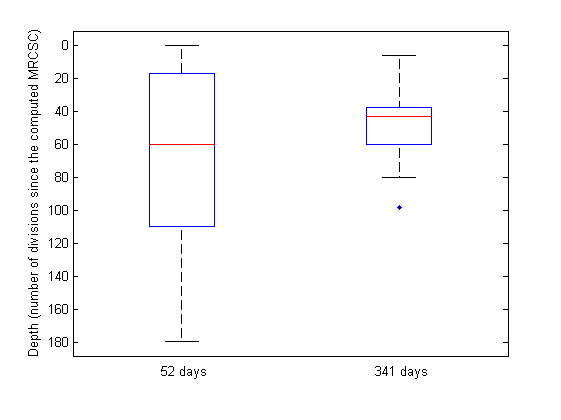

Supplement: Figure S3 — No statistically significant difference in relative depth, between young and old mice. Box plots of the relative depths between individual crypt cells to their computed MRCSCs in 52 and 340 day-old mice. There is no statistically significant difference in relative depth between 52 and 340 day-old mice. (TIF) [file pgen.1002192.s003.tif]

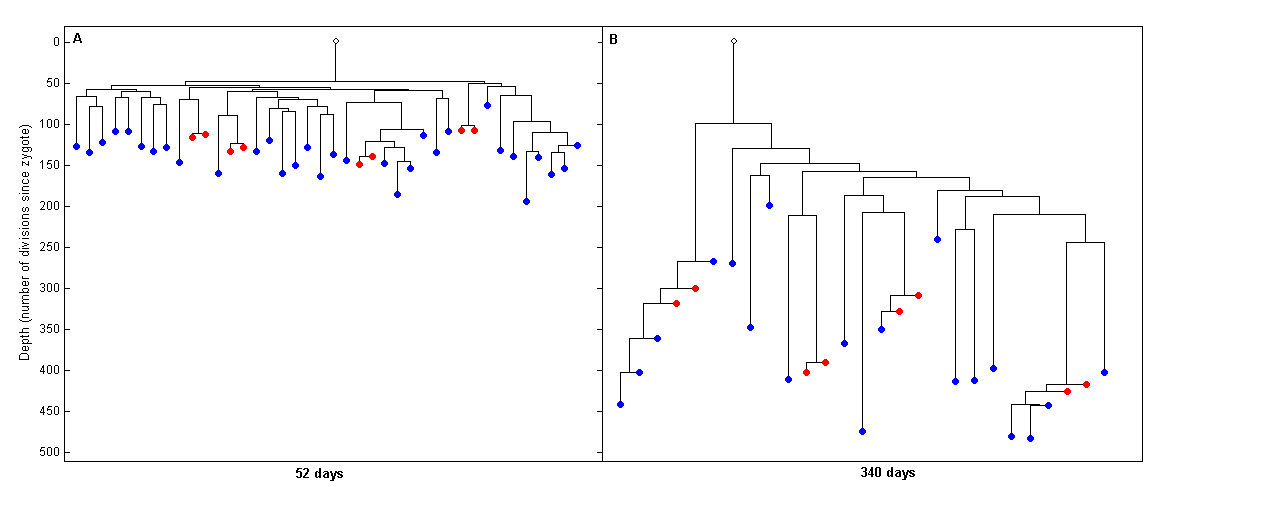

Supplement: Figure S4 — PCR repeats are clustered on the reconstructed lineage tree. Reconstructed lineage tree of 52 and 340 day-old mice (Figure 3A and 3B), shows that PCR repeats (red) are strongly clustered and share similar depths. Similar results were received in all trees. (TIF) [file pgen.1002192.s004.tif]

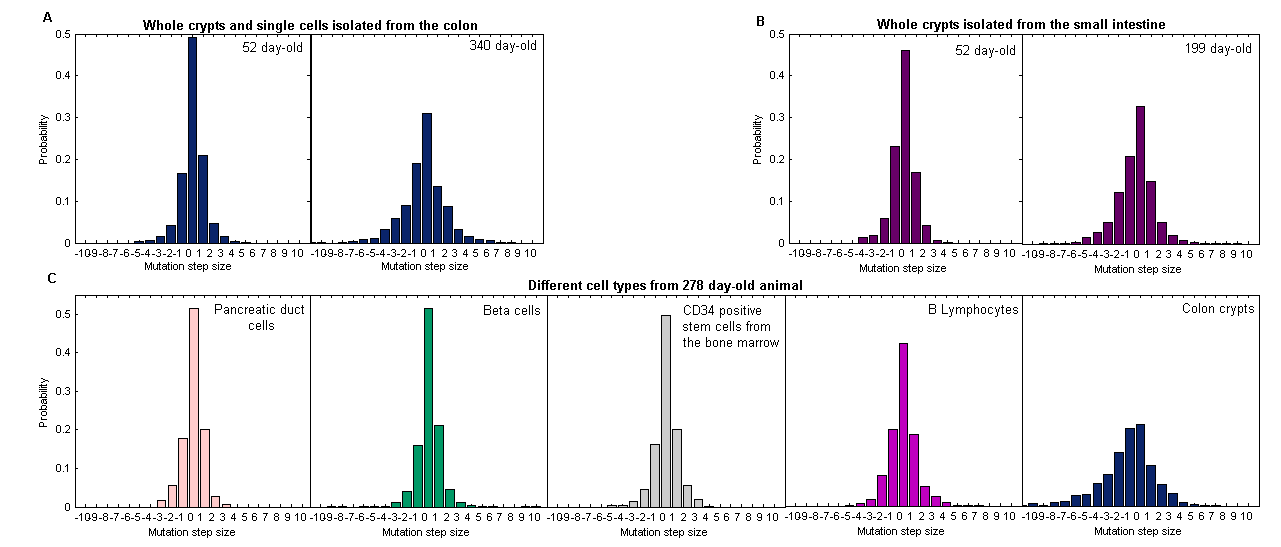

Supplement: Figure S5 — Mutation size frequencies in different cell types from different mice. (A), Mutation size frequencies of whole crypts and single cells isolated from the colon of 52 and 340 day-old mice. (B), Mutation size frequencies of whole crypts isolated from small intestine of 52 and 199 day-old mice. In both A and B, the mutation step size distribution is much wider in the older animal, showing positive correlation between number of cell divisions and mutation lengths. (C), Mutation size frequencies of different cell types from 278 day-old mouse. The distribution of the colon crypts is the widest among all the cell types presented in this panel, since the colon crypts accumulated more MS mutations. (TIF) [file pgen.1002192.s005.tif]
